# From disease activity scores to precision medicine: biomarkers and composite models for risk stratification in chronic spontaneous urticaria

**DOI:** 10.3389/falgy.2026.1962595

**Published:** 2026-10-08

**Authors:** Shuayb Elkhalifa, Mohamed Abuzakouk

**Affiliations:** 1 Allergy and Immunology Department, Medical Subspecialties Institute, Cleveland Clinic Abu Dhabi, Abu Dhabi, United Arab Emirates; 2 Centre for Musculoskeletal Research, Faculty of Biology, Medicine and Health, The University of Manchester, United Kingdom

**Keywords:** angioedema, biomarkers, chronic spontaneous urticaria (CSU), dubilumab, omalizumab, precision medicine, prognosis, remibrutinib

## Abstract

Chronic spontaneous urticaria is a common, mast cell driven condition that presents with wheals, angioedema, or both, and a substantial proportion of patients remain inadequately controlled despite guideline-directed antihistamine therapy. The 2026 guideline has expanded the advanced-treatment options to include omalizumab, dupilumab and remibrutinib, while ciclosporin remains an alternative later-line immunosuppressive option, yet it is organised around treatment response rather than prognosis, so patients with difficult-to-control disease are often recognised only after months of sequential escalation. This narrative review examines the scoring systems and biomarkers available in urticaria and angioedema and considers whether they can support early risk stratification and help identify patients at increased risk of difficult-to-control disease. The validated instruments in routine use, namely the Urticaria Activity Score, the Urticaria Control Test, the Chronic Urticaria Quality of Life Questionnaire, and their angioedema counterparts, measure current disease activity, control, and quality of life rather than long term outcome. A separate body of work has described biomarkers associated with the autoimmune type IIb endotype and with response to omalizumab, including low total IgE, a high ratio of IgG anti-thyroid peroxidase to total IgE, basopenia, and raised inflammatory markers, although most of this evidence is associative rather than prospectively predictive. The distinction between markers of current burden, markers of prognosis, and markers of treatment response is emphasised. Composite models integrating clinical characteristics, patient-reported outcomes, and biomarkers represent a promising approach toward precision management of CSU, although no validated prognostic tool is currently available for routine clinical use.

## Key points

1. Chronic spontaneous urticaria has an unpredictable course, and a substantial proportion of patients remain inadequately controlled despite guideline-directed antihistamine therapy. Because current algorithms are organised around treatment response rather than prognosis, difficult-to-control disease is often recognised only after prolonged and unsuccessful escalation.
2. This review distinguishes validated instruments that monitor current disease activity, control, and quality of life from biomarkers that reflect the autoimmune type IIb endotype or are associated with treatment response, and it emphasises that most of these biomarkers are associative rather than validated predictive tools.
3. No validated composite model yet identifies patients at increased risk of difficult-to-control disease at presentation. Composite models integrating clinical characteristics, patient-reported outcomes, and biomarkers are a promising research direction, and the CLARITY framework is presented as one investigational example.

## Introduction

Chronic spontaneous urticaria (CSU) is defined by the recurrence of wheals, angioedema, or both for more than 6 weeks without a consistent external trigger. It is common, it is driven by the activation of skin mast cells, and it carries a quality of life burden comparable with ischaemic heart disease (1, 2). The 2026 guideline recommends a stepwise approach that begins with a second generation H1 antihistamine, escalates to a fourfold dose, and then expanded the advanced-treatment options to include omalizumab, dupilumab and remibrutinib, while ciclosporin remains an alternative later-line immunosuppressive (1, 2). Omalizumab has transformed the care of antihistamine refractory disease, dupilumab has shown efficacy irrespective of a low baseline total immunoglobulin E (IgE) level, and remibrutinib, an oral Bruton tyrosine kinase inhibitor, is effective independent of previous omalizumab use (3, 4).

These algorithms are organised around treatment response rather than prognosis. A substantial proportion of patients remain inadequately controlled despite guideline-directed antihistamine therapy, and difficult-to-control disease is frequently recognised only after months of sequential escalation. There is therefore an unmet need for tools that allow early risk stratification, so that patients at increased risk of a difficult course can be recognised sooner. Clinical observations and emerging evidence suggest that difficult-to-control CSU may cluster with identifiable clinical and immunological characteristics; however, these features have not yet been integrated into validated predictive models (see box 1). This article reviews the instruments and biomarkers available in urticaria and angioedema, considers what each can and cannot do, and asks whether they can support early risk stratification and, ultimately, the earlier consideration of advanced therapies (see box 2).

Box 1 **Practical summary**

**Table:** 

- Validated instruments (UAS7, UCT, CU-Q2oL, AAS, AE-QoL, AECT) measure current activity, control, and quality of life, not prognosis.
- Biomarkers of the type IIb endotype and of omalizumab response are largely associative, are not yet validated predictors, and may not apply to dupilumab or remibrutinib.
- Monitoring, prognostic, and predictive markers, and the outcomes of disease persistence, severity, and treatment response, should be kept conceptually separate.
- No validated composite prognostic model exists. Investigational frameworks such as CLARITY require prospective and external validation before clinical use.

Box 2 **Key unanswered questions**

**Table:** 

- Which biomarkers are sufficiently reliable for routine practice?
- Can composite models outperform single biomarkers?
- Should prediction models be therapy-specific?
- Can early risk stratification improve patient outcomes?
- Is biomarker-guided treatment cost-effective?

This narrative review was based on a structured search of PubMed, Embase, and recent international guidelines using terms related to chronic spontaneous urticaria, biomarkers, endotypes, prognosis, omalizumab, dupilumab, remibrutinib, and biologics. Priority was given to systematic reviews, international guidelines, prospective cohort studies, and major clinical trials.

For clarity, three terms are used throughout with distinct meanings. Difficult-to-control CSU refers to disease that remains inadequately controlled, for example a UAS7 in the moderate to severe range or a UCT below 12, despite guideline-directed therapy up to and including high-dose antihistamines and omalizumab. Disease persistence refers to a long disease duration and a failure to remit over time. Response to treatment refers to the achievement of disease control on a specified therapy. These outcomes are related but distinct, and a prognostic tool and a predictive tool address different ones. A prognostic tool estimates the risk of a difficult future course, whereas a predictive tool estimates the likelihood of response to a specific therapy. The CLARITY framework discussed below is a prognostic, risk-stratification framework. It is not a diagnostic test, and in its current form it is not a predictive or treatment decision tool (see box 1 and box 2).

## Instruments that measure activity, control, and quality of life

Several validated patient reported instruments are recommended by the guideline for routine use, and each has a clear and specific purpose (1). The Urticaria Activity Score summed over 7 days (UAS7) is the most widely used measure of disease activity. It sums a daily record of wheal number and itch intensity, ranges from 0 to 42, and has been shown to be valid, reliable, and responsive to change (5). Scores of 7 to 15, 16 to 27, and 28 to 42 correspond to mild, moderate, and severe activity. The Urticaria Control Test is a four item retrospective questionnaire that captures disease control over recent weeks, with a score of 12 or more indicating well controlled disease (6). The Chronic Urticaria Quality of Life Questionnaire is a 23 item disease specific measure of the impact of urticaria on daily life (7).

For patients whose presentation is dominated by angioedema, parallel instruments exist. The Angioedema Activity Score measures the activity of recurrent swelling, the Angioedema Quality of Life Questionnaire measures its impact, and the Angioedema Control Test measures control, with a cutoff of 10 or more identifying well controlled disease (8–10). Together these instruments give a rounded picture of the burden of disease and are essential for monitoring the response to treatment over time. Their limitation, for the present purpose, is that they were designed to quantify the current state of disease rather than to predict its course. A high UAS7 at baseline is associated with a lower probability of early remission, so activity carries some prognostic weight, but none of these measures incorporates the immunological markers that distinguish the endotypes of CSU, and none was designed to stratify, at diagnosis, the risk of a difficult course. These instruments quantify current disease burden and are intentionally responsive to changes over time. They were not developed using longitudinal outcome modelling, they do not incorporate biological variables, and they therefore cannot distinguish patients with transient severe disease from those with persistent difficult-to-control disease. Table 1 summarises the instruments and what each is designed to measure.

**Table 1:** Validated patient reported instruments used to monitor chronic urticaria and angioedema.

Instrument	What it measures	Format	Role
UAS7	Disease activity (wheals and itch) over 7 days	Daily patient diary, score 0 to 42	Monitoring
UCT	Disease control over recent weeks	Four items, score 0 to 16	Monitoring
CU-Q2oL	Quality of life impact of urticaria	23 items	Monitoring
AAS	Recurrent angioedema activity	Patient diary	Monitoring
AE-QoL	Quality of life impact of angioedema	17 items	Monitoring
AECT	Angioedema control	Four items, score 0 to 16	Monitoring

AAS, angioedema activity score; AECT, angioedema control test; AE-QoL, angioedema quality of life questionnaire; CU-Q2oL, chronic urticaria quality of life questionnaire; UAS7, urticaria activity score over 7 days; UCT, urticaria control test.

## Biomarkers of endotype and treatment response

A second and largely separate body of work has sought biomarkers that help to explain why patients differ in their course and in their response to treatment. Much of it centres on the autoimmune, or type IIb, endotype. In approximately 40% of patients the inflammation is driven by autoantibodies, either immunoglobulin E autoantibodies against self antigens in type I autoallergic disease or immunoglobulin G autoantibodies that activate mast cells in type IIb disease, and many patients show features of both (11, 12). In research settings the type IIb endotype is supported by a positive autologous serum skin test, a positive basophil activation test, and autoantibodies against IgE or its high affinity receptor. In routine practice it can be approximated by a cluster of accessible findings, namely a low total IgE, an elevated IgG anti-thyroid peroxidase, basopenia, and eosinopenia (13).

These markers are of interest because they are associated with a poorer response to omalizumab. A low baseline total IgE is associated with coexisting autoimmune disease and with a slower and only partial response, and a high ratio of IgG anti-thyroid peroxidase to total IgE, above a reported cutoff of 0.154, was associated with roughly tenfold higher odds of a poor response in the original study (14, 15). The value of 0.154 refers to the ratio of IgG anti-thyroid peroxidase to total IgE reported by Bras and colleagues, and not to a total IgE concentration (15). Reported thresholds for the individual markers vary between studies; low total IgE cutoffs have ranged from approximately 40 to 44 IU/mL, and the optimal value for the ratio has not been established and requires prospective confirmation (14). The guideline now recommends that patients in specialist care have total IgE and IgG anti-thyroid peroxidase measured alongside a differential blood count and C-reactive protein, and it identifies the ratio of IgG anti-thyroid peroxidase to total IgE as the best current surrogate for the type IIb endotype (1, 16). Inflammatory markers add further information, since a raised C-reactive protein, a raised D-dimer, and reduced basophil and eosinophil counts have been linked with more active disease and with a slower response (16, 17). It is important to note that most of these markers are associated with disease severity, immunological endotype, or a delayed or reduced response, rather than being validated predictors of treatment failure, and few have undergone prospective validation.

Attempts to combine these signals are at an early stage. Real world analyses have tested panels of serum biomarkers for their association with the response to omalizumab, and an exploratory machine learning study used baseline variables, including D-dimer and C-reactive protein, to classify patients as early, late, or non responders (17, 18). These studies suggest that risk stratification is possible in principle, but they also show that no single marker, and no published model, is yet accurate or validated enough for routine use.

Much of this literature has focused on the response to omalizumab. Biomarkers that predict the response to omalizumab may not predict the response to dupilumab or remibrutinib, and future prediction models will probably require treatment-specific validation as therapeutic targets become increasingly diverse.

## Distinguishing monitoring, prognostic, and predictive markers

A recurring source of confusion is the conflation of markers that answer different questions, and keeping them separate is essential both when interpreting the literature and when designing composite models. Monitoring tools answer the question of how active or controlled the disease is at present, and comprise the patient reported instruments described above. Prognostic markers answer the question of who is likely to have persistent or difficult-to-control disease, and include a long disease duration, high baseline activity, angioedema, coexisting chronic inducible urticaria, and the autoimmune phenotype. Predictive biomarkers answer the question of who is likely to respond to a specific therapy, and include the baseline total IgE and the type IIb autoimmune markers. It is equally important to distinguish the outcomes themselves, since disease persistence, disease severity, and poor treatment response are related but distinct, and a prediction model must specify which it targets. Figure 1 sets out these distinctions.

**Figure 1:**
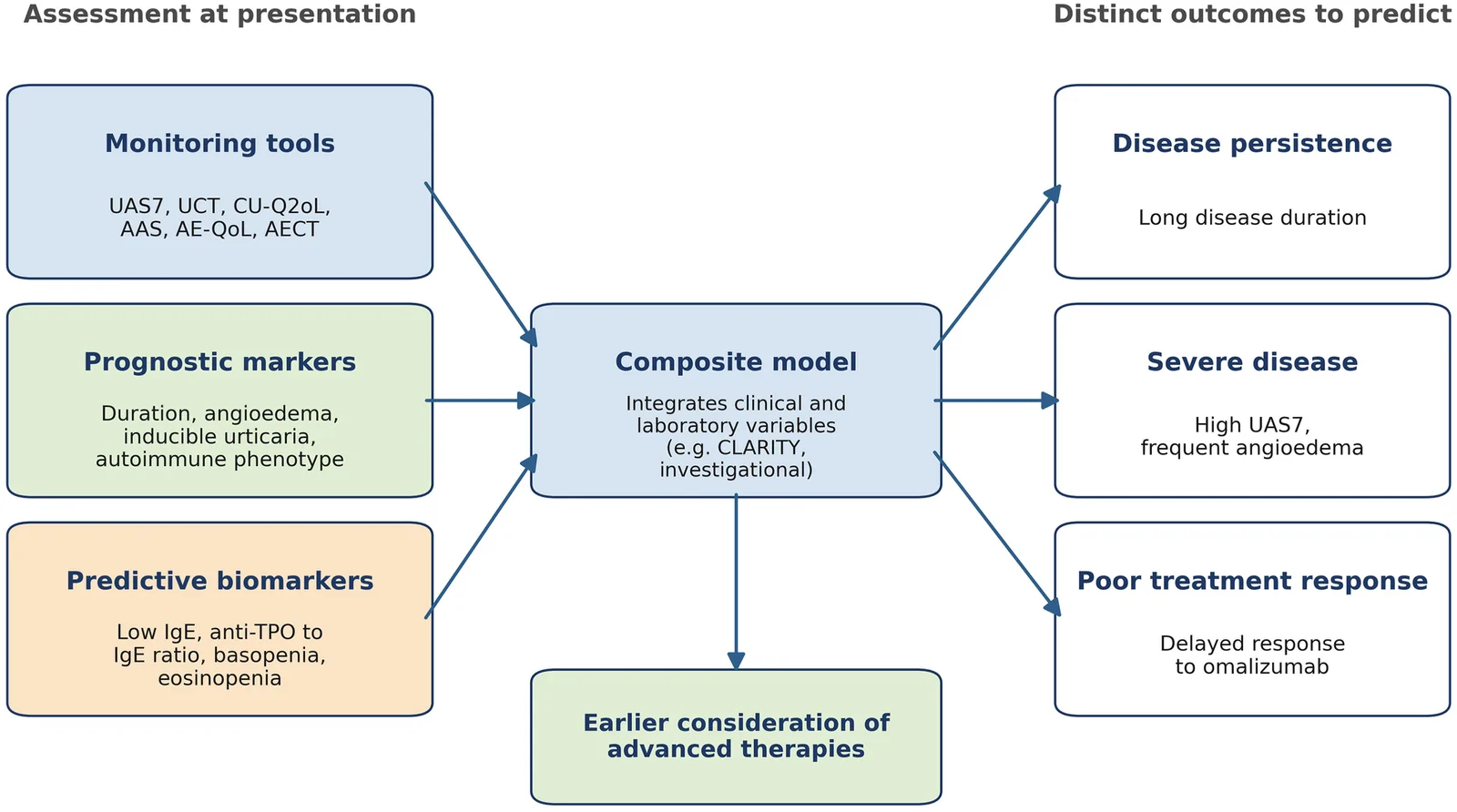
Conceptual framework for risk stratification in chronic spontaneous urticaria. Monitoring tools, prognostic markers, and predictive biomarkers assessed at presentation address three related but distinct outcomes, namely disease persistence, disease severity, and poor treatment response. These signals may be integrated into a composite model, such as the investigational CLARITY framework, to support the earlier consideration of advanced therapies, an approach that has not yet been shown to modify the natural history and requires prospective testing. Figure uploaded separately.

## Prognostic factors and natural history

Prognosis in CSU is heterogeneous. Roughly one third to one half of patients remit within a year, yet a meaningful proportion continue with active disease at 5 years. Factors associated with a longer course include a younger age at onset, a relapsing and remitting pattern, the presence of angioedema, high disease activity, coexisting chronic inducible urticaria, and functional serum autoreactivity (19). Studies of antihistamine refractory cohorts have identified angioedema, high baseline activity, and autoimmune comorbidity among the factors associated with poor control (20). Thyroid autoimmunity is the most consistent comorbid signal, since thyroid autoantibody positivity is associated with a longer disease duration, greater severity, and a lower response rate at week 24 of treatment (21). These observations show that prognostic information is scattered across separate variables and separate studies, and that it has not yet been drawn together into a validated instrument.

## Predicting response to dupilumab and remibrutinib

The biomarker literature summarised above concerns the response to omalizumab, and its conclusions should not be extrapolated to the newer agents. Dupilumab blocks interleukin 4 and interleukin 13 signalling and is effective irrespective of the baseline total IgE, so its benefit may be greater in patients with a type 2 inflammatory profile or atopic comorbidity, although validated predictors of response are not yet available (3). Remibrutinib inhibits Bruton tyrosine kinase and acts downstream of the high affinity IgE receptor on mast cells and basophils, and it is effective independently of the baseline IgE and of previous omalizumab use, which suggests activity across endotypes, including in patients who have failed anti-IgE therapy (4). Direct comparisons of the three agents are only beginning to appear, and the factors that determine refractoriness to dupilumab or to remibrutinib remain undefined (22). Prediction in CSU will therefore need to be developed and validated separately for each agent rather than assumed to be shared, since a biomarker useful for one mechanism may be uninformative for another.

## The case for composite models

Two conclusions follow. First, the instruments used every day measure the present state of disease and were not designed to predict its course. Second, the biomarkers that carry prognostic or predictive information are informative individually but modest on their own, and they are not yet combined into a validated tool. The updated guideline recognised this directly, since it introduced, for the first time, a dedicated question on biomarkers that predict the disease course, which places the prediction of prognosis on the formal research agenda (1). A composite model that integrates a measure of disease activity, the biomarkers of the type IIb endotype, and the relevant clinical factors could, in principle, support earlier and more individualised decisions, including the earlier consideration of advanced therapies. The clinical need is considerable, since patients with difficult-to-control disease accumulate the greatest burden of symptoms, corticosteroid exposure, and healthcare use during the period before effective treatment is reached (20). Integer point scores derived from multivariable models are used routinely for risk stratification in other fields of medicine, which supports the feasibility of an analogous approach in CSU once adequate prospective data are available (23). Any such model must, however, define its target outcome. Disease persistence, disease severity, and poor treatment response are distinct, and for the framework described below the primary endpoint of the planned validation is difficult-to-control disease, defined as failure of high-dose antihistamine therapy and a requirement for advanced therapy, with disease persistence at 12 months and response to specific agents examined as separate secondary outcomes.

## Towards composite prognostic models in CSU: the CLARITY framework as an example

Current evidence provides a strong rationale for the development and prospective validation of composite prognostic models, and several are likely to emerge. The CLARITY framework is presented here as one example of how clinical and laboratory variables could be combined, rather than as a validated clinical instrument. As set out above, it is a prognostic, risk-stratification framework that estimates the risk of a difficult course, and not a predictive tool for the response to a particular therapy nor a treatment decision instrument. It is a weighted framework in which each of seven domains, captured by the letters of its name, scores one point, giving a total of 0 to 7. The domains span an immunological component, namely a low total IgE and a type IIb autoimmune signature, and a clinical component, namely comorbid autoimmune disease, recurrent angioedema, coexisting inducible urticaria, treatment burden with high disease activity, and symptom duration before referral. Only variables with published evidence of an association with disease persistence, severity, or treatment response have been included, and each is defined so that it is available at the first specialist assessment. Earlier candidate domains based on complement abnormalities and basal tryptase have been removed because the supporting evidence in CSU is insufficient. The domains, their definitions, and the supporting references are shown in Table 2.

**Table 2:** The CLARITY framework, presented as an investigational composite model.

Letter	Domain	Pillar	Pts	Definition	Evidence
C	Comorbid autoimmune disease	Clinical	1	Active autoimmune or autoinflammatory disease, for example autoimmune thyroid disease	(21)
L	Low total IgE	Immunological	1	Low or very low baseline total IgE, associated with the type IIb endotype and a slower or reduced omalizumab response	(14, 15)
A	Autoimmune type IIb signature	Immunological	1	Elevated IgG anti-TPO with a high IgG anti-TPO to total IgE ratio (for example above 0.154), supported by basopenia and eosinopenia and by positive ASST or BAT where available	(11, 12, 13, 16)
R	Recurrent angioedema	Clinical	1	Recurrent angioedema as part of the presentation	(19, 20)
I	Inducible urticaria	Clinical	1	Coexisting chronic inducible urticaria, associated with greater disease burden	(19)
T	Treatment burden and high disease activity	Clinical	1	High baseline activity (for example UAS7 above 28), previous systemic corticosteroid courses, and failure of high-dose antihistamine therapy	(19, 20)
Y	Years: symptom duration before referral	Clinical	1	Symptom duration greater than 12 months before specialist referral, a clearly defined baseline characteristic available at first assessment	(19)
	Maximum total score		7		

Evidence refers to the numbered references in this article.

anti-TPO, anti-thyroid peroxidase antibody; ASST, autologous serum skin test; BAT, basophil activation test; IgE, immunoglobulin E; IgG, immunoglobulin G; UAS7, urticaria activity score over 7 days.

Each of seven evidence-based domains scores one point, giving a total of 0 to 7. The framework has not been validated.

All domains currently carry equal weight, that is, each contributes one point. Equal, or unit, weighting was chosen deliberately. In the absence of prospective outcome data from which to estimate coefficients, unit weighting is a recognised and well-established approach for a provisional risk score, since simple integer point systems perform comparably to optimally weighted models when the derivation sample is limited, and it reduces the risk of overfitting (23). The weighting is therefore provisional, based on biological plausibility and on the consistency of the reported associations in the literature, and domain-specific weights will be estimated by regression during the planned derivation and validation studies. In this illustrative scheme, a higher total would flag a greater likelihood of difficult-to-control disease and could support individualised treatment escalation following prospective validation, while a lower total would support standard stepwise care. The framework is intended to generate hypotheses and to structure future research. It is investigational and should not be used as a clinical decision-making tool until external validation has been completed.

The CLARITY framework is currently under investigation. A retrospective cohort study at the authors' centre is scoring the domains from records available at first presentation and relating them to the subsequent response to antihistamines and to advanced therapy, the time to control, corticosteroid exposure, and remission, with internal validation by resampling. A prospective study is planned to refine the weighting, calibrate the thresholds, and measure discriminative performance. The full development and validation will be reported separately. Until prospective and external validation are available, the framework should be regarded as hypothesis generating and should not guide individual treatment decisions.

## Limitations

Several limitations apply to the current evidence and to composite models in this field. Existing prediction models lack prospective validation and external validation. Most biomarker studies are observational and involve relatively small cohorts, which limits the certainty of the reported associations across different healthcare settings. Definitions of refractory or difficult-to-control CSU are heterogeneous, and treatment response endpoints vary between studies, which limits comparison. Much of the supporting evidence derives from tertiary referral populations and is therefore subject to referral bias, with limited applicability to community-based populations. Taken together, these constraints mean that current biomarkers and composite frameworks provide biological and clinical insight but are not yet ready to guide routine care, which limits their clinical applicability and underlines the need for prospective, externally validated studies.

## Future directions

Progress will depend on several parallel efforts: the prospective validation of candidate biomarkers; multi-centre international validation across different populations and healthcare settings; the integration of clinical and laboratory variables into composite models; the incorporation of genomic, transcriptomic, and proteomic data; the use of machine-learning approaches; the prediction of response to specific agents, including omalizumab, dupilumab, and remibrutinib; and the health-economic evaluation of risk-stratified treatment pathways. Advances in these areas would move the field from the description of associations toward validated tools that can be used with confidence in routine care.

A related but separate question is whether the transition from acute to chronic urticaria can be predicted. The CLARITY framework applies only to patients already diagnosed with CSU and does not address this transition. A proportion of acute urticaria progresses to chronic disease, and a positive autologous serum skin test, thyroid autoimmunity, and basopenia at presentation have been associated with a higher risk of progression, but no predictor is yet reliable enough for clinical use, and this remains a distinct avenue for future research (24).

## Conclusion

CSU management currently benefits from validated tools that assess disease activity, control, and quality of life, and from an expanding understanding of immunological endotypes. However, validated prognostic models capable of identifying patients at increased risk of difficult-to-control disease remain unavailable. Composite models integrating clinical characteristics, patient-reported outcomes, and biomarkers represent a rational next step toward personalised CSU management, and prospective validation will determine their future clinical utility.

## Disclosure

SE has received research funding from Health Education England and Manchester University NHS Foundation Trust, has acted as an expert advisor for MTEP and NICE, and has taken part in advisory boards, expert consultations, or received travel or speaking honoraria from AstraZeneca, Sanofi, Novartis, GSK, Biologix, Newbridge, BioCryst, CSL, and Takeda. MA: disclosures to be confirmed by the author. None of the disclosed relationships relate to the content of this review.

## Data Availability

The original contributions presented in the study are included in the article/Supplementary Material, further inquiries can be directed to the corresponding author.

## References

[1] Zuberbier T Ansari ZAH Abdul Latiff AH Abuzakouk MM Agcaoili-De Jesus MS Agondi RC. The international guideline for the definition, classification, diagnosis and management of urticaria. Allergy. (2026) 81:2582–632. 10.1111/all.70210 41649409 PMC13466004

[2] Zuberbier T Abdul Latiff AH Abuzakouk M Aquilina S Asero R Baker D. The international EAACI/GA2LEN/EuroGuiDerm/APAAACI guideline for the definition, classification, diagnosis and management of urticaria. Allergy. (2022) 77(3):734–66. 10.1111/all.15090 34536239

[3] Maurer M Casale TB Saini SS Ben-Shoshan M Giménez-Arnau AM Bernstein JA. Dupilumab in patients with chronic spontaneous urticaria (LIBERTY-CSU CUPID): two randomized, double-blind, placebo-controlled, phase 3 trials. J Allergy Clin Immunol. (2024) 154(1):184–94. 10.1016/j.jaci.2024.01.028 38431226

[4] Metz M Giménez-Arnau A Hide M Lebwohl M Mosnaim G Saini S. Remibrutinib in chronic spontaneous urticaria. N Engl J Med. (2025) 392(10):984–94. 10.1056/NEJMoa2408792 40043237

[5] Hawro T Ohanyan T Schoepke N Metz M Peveling-Oberhag A Staubach P. The urticaria activity score: valid, reliable, and responsive. J Allergy Clin Immunol Pract. (2018) 6(4):1185–90. 10.1016/j.jaip.2017.10.001 29128337

[6] Weller K Groffik A Church MK Hawro T Krause K Metz M. Development and validation of the urticaria control test: a patient-reported outcome instrument for assessing urticaria control. J Allergy Clin Immunol. (2014) 133(5):1365–72. 10.1016/j.jaci.2013.12.1076 24522090

[7] Baiardini I Pasquali M Braido F Fumagalli F Guerra L Compalati E. A new tool to evaluate the impact of chronic urticaria on quality of life: chronic urticaria quality of life questionnaire (CU-Q2oL). Allergy. (2005) 60(8):1073–8. 10.1111/j.1398-9995.2005.00833.x 15969690

[8] Weller K Groffik A Magerl M Tohme N Martus P Krause K. Development, validation, and initial results of the angioedema activity score. Allergy. (2013) 68(9):1185–92. 10.1111/all.12209 23919330

[9] Weller K Groffik A Magerl M Tohme N Martus P Krause K. Development and construct validation of the angioedema quality of life questionnaire (AE-QoL). Allergy. (2012) 67(10):1289–98. 10.1111/all.12007 22913638

[10] Weller K Donoso T Magerl M Aygören-Pürsün E Staubach P Martinez-Saguer I. Validation of the angioedema control test (AECT): a patient-reported outcome instrument for assessing angioedema control. J Allergy Clin Immunol Pract. (2020) 8(6):2050–7. 10.1016/j.jaip.2020.02.038 32173507

[11] Schoepke N Asero R Ellrich A Ferrer M Gimenez-Arnau A Grattan CEH. Biomarkers and clinical characteristics of autoimmune chronic spontaneous urticaria: results of the PURIST study. Allergy. (2019) 74(12):2427–36. 10.1111/all.13949 31228881

[12] Kolkhir P Muñoz M Asero R Ferrer M Kocatürk E Metz M. Autoimmune chronic spontaneous urticaria. J Allergy Clin Immunol. (2022) 149(6):1819–31. 10.1016/j.jaci.2022.04.010 35667749

[13] Sella JA Ferriani MPL Melo JML Trevisan Neto O Zanetti MET Cordeiro DL. Type I and type IIb autoimmune chronic spontaneous urticaria: using common clinical tools for endotyping patients with CSU. J Allergy Clin Immunol Glob. (2023) 2(4):100159. 10.1016/j.jacig.2023.100159 37781653 PMC10509944

[14] Pesqué D March-Rodríguez Á Curto-Barredo L Soto D Gimeno R Pujol RM. Autoimmune diseases and low baseline IgE in chronic spontaneous urticaria: a clinical and therapeutic prospective analysis in real-life clinical practice. J Allergy Clin Immunol Pract. (2023) 11(12):3763–72. 10.1016/j.jaip.2023.09.002 37716526

[15] Bras R Esteves Caldeira L Bernardino A Costa C. Anti-TPO IgG/total IgE ratio: biomarker for omalizumab response prediction in chronic spontaneous urticaria. Int Arch Allergy Immunol. (2023) 184(9):866–9. 10.1159/000532021 37557083

[16] Lang DM Sheikh J Joshi S Bernstein JA. Endotypes, phenotypes, and biomarkers in chronic spontaneous urticaria: Evolving toward personalized medicine. Ann Allergy Asthma Immunol. (2025) 134(4):408–17. 10.1016/j.anai.2024.10.026. 39490777

[17] Cain WV Jandarov RA Priya M Rao M Bernstein JA. Utility of serum biomarkers in real-world practice for predicting response to omalizumab therapy in patients with chronic spontaneous urticaria. J Allergy Clin Immunol Glob. (2024) 3(2):100234. 10.1016/j.jacig.2024.100386 39844914 PMC11751516

[18] Sardina DS Valenti G Papia F Uasuf CG. Exploring machine learning techniques to predict the response to omalizumab in chronic spontaneous urticaria. Diagnostics (Basel). (2021) 11(11):2150. 10.3390/diagnostics11112150 34829497 PMC8623518

[19] Stepaniuk P Kan M Kanani A. Natural history, prognostic factors and patient perceived response to treatment in chronic spontaneous urticaria. Allergy Asthma Clin Immunol. (2020) 16:63. 10.1186/s13223-020-00459-5 32834828 PMC7371813

[20] Alen Coutinho I Regateiro FS Fernandes RA Pita JS Gomes R Coelho C. Refractory chronic urticaria in adults: clinical characterization and predictors of severity. Allergy Asthma Clin Immunol. (2020) 16:97. 10.1186/s13223-020-00496-0 33292453 PMC7661155

[21] Chanprapaph K Iamsumang W Wattanakrai P Vachiramon V. Thyroid autoimmunity and autoimmunity in chronic spontaneous urticaria linked to disease severity, therapeutic response, and time to remission. Biomed Res Int. (2018) 2018:9856843–13. 10.1155/2018/9856843 30515422 PMC6236973

[22] Türk M Yılmaz İ Ertaş R Kocatürk E. Efficacy of omalizumab, dupilumab, and remibrutinib in chronic spontaneous urticaria: phase data and placebo response. Allergy. (2025) 80:2410–3. 10.1111/all.16670 40682317

[23] Sullivan LM Massaro JM D'Agostino RB Sr. Presentation of multivariate data for clinical use: the framingham study risk score functions. Stat Med. (2004) 23(10):1631–60. 10.1002/sim.1742 15122742

[24] Magen E Zueva E Mishal J Schlesinger M. The clinical and laboratory characteristics of acute spontaneous urticaria and its progression to chronic spontaneous urticaria. Allergy Asthma Proc. (2016) 37(5):394–9. 10.2500/aap.2016.37.3971 27657523

